# Identification of new signalling peptides through a genome-wide survey of 250 fungal secretomes

**DOI:** 10.1186/s12864-018-5414-2

**Published:** 2019-01-18

**Authors:** Morgane Le Marquer, Hélène San Clemente, Christophe Roux, Bruno Savelli, Nicolas Frei dit Frey

**Affiliations:** Laboratoire de Recherche en Sciences Végétales, Université de Toulouse, CNRS, UPS, 24 chemin de Borde Rouge, Auzeville, BP42617, 31326 Castanet Tolosan, France

**Keywords:** KEX2, Secreted peptide, Mycotoxin, Effector, Fungi, Secretome, Sexual pheromone

## Abstract

**Background:**

Many small peptides regulate eukaryotic cell biology. In fungi, some of these peptides are produced after KEX2 protease activity on proteins displaying repetitions of identical or nearly identical motifs. Following this endoprotease activity, peptides are released in the extracellular space. This type of protein maturation is involved in the production of the α-type sexual pheromone in Ascomycota. In other cases, this processing allows the production of secreted peptides regulating fungal cell wall structure or acting as mycotoxins. In this work, we report for the first time a genome-wide search of KEX2-processed repeat proteins that we call KEPs. We screened the secreted proteins of 250 fungal species to compare their KEP repertoires with regard to their lifestyle, morphology or lineage.

**Results:**

Our analysis points out that nearly all fungi display putative KEPs, suggesting an ancestral origin common to all opisthokonts. As expected, our pipeline identifies mycotoxins but also α-type sexual pheromones in Ascomycota that have not been explored so far, and unravels KEP-derived secreted peptides of unknown functions. Some species display an expansion of this class of proteins. Interestingly, we identified conserved KEPs in pathogenic fungi, suggesting a role in virulence. We also identified KEPs in Basidiomycota with striking similarities to Ascomycota α-type sexual pheromones, suggesting they may also play alternative roles in unknown signalling processes.

**Conclusions:**

We identified putative, new, unexpected secreted peptides that fall into different functional categories: mycotoxins, hormones, sexual pheromones, or effectors that promote colonization during host-microbe interactions. This wide survey will open new avenues in the field of small-secreted peptides in fungi that are critical regulators of their intimate biology and modulators of their interaction with the environment.

**Electronic supplementary material:**

The online version of this article (10.1186/s12864-018-5414-2) contains supplementary material, which is available to authorized users.

## Background

Secreted signalling peptides regulate crucial cell activities in opisthokonts. In humans, peptide hormones control pain, appetite, bone formation, digestion, glucose homeostasis or the maturation of sexual organs. Some of these peptides have a very small size such as substance P (11 amino acids (aa)) or thyrotropin releasing hormone (3 aa) [1]. Secreted peptides also regulate many aspects of fungal biology. In Ascomycota, mating is preceded by the perception of the α- and the a-type pheromones [2]. The α pheromone is produced from prepro-proteins MF(ALPHA)1 and MF(ALPHA)2 in *Saccharomyces cerevisiae*. These two proteins display a signal peptide, a pro-region and four (MF(ALPHA)1) or two (MF(ALPHA)2) repetitions of nearly identical motifs. Each motif is preceded by an easily recognizable protease cleavage site. This cleavage site is composed of the “KR” dipeptide followed by “EA” or “DA” dipeptides (e.g. KREA or KRDA). The KEX1 and KEX2 proteases respectively cut before the K and after the R of the KR dipeptide [3, 4]. STE13 then cleaves after the A of the EA or DA residues, thus releasing the repeated peptides. This processing occurs in the Golgi apparatus while the protein passes through the secretory pathway. Peptides are finally released in the extracellular space by exocytosis and do not present post-translational modifications. Among fungi, the KEX2 cleavage site is highly conserved and generally consists of the dipeptide KR or RR, and to a lesser extent KK [5]. The motif recognized by STE13 is however more variable in sequence and in length and is often a repetition of XA or XP dipeptides (X: any amino acid) [3, 6, 7]. An extended survey of Ascomycota α pheromone encoding genes revealed that the number of repeated peptides is also very variable across Ascomycota, ranging from one (e.g. *Saccharomyces castellii*) to 16 secreted peptides (e.g. *Fusarium subglutinans)* [8]. It also revealed that the average number of repeats was five, that the size of the secreted peptides was ranging from nine (e.g. Aspergillus species) to 24 amino acids (e.g. Schyzosaccharomyces species) and that sequence variation was frequent among the different repeats of a single precursor protein.

These α pheromone precursors display an original and inventive way to produce large amounts of repeated peptides originating from a single transcript. Interestingly, other types of peptides, with no link with sexuality, are produced in the same way. In Basidiomycota for example, the plant pathogen *Ustilago maydis* secretes 37-aa-long peptides after cleavage of the Rep1 precursor protein [9]. These peptides control *U. maydis* hyphal wall properties. In Ascomycota, cyclic and modified peptides are released after KEX2 processing of precursor proteins. It is the case in Epichloë endophytes of grasses [10], but also in larger groups where they act as mycotoxins [5, 11]. Gaining knowledge on the potential of fungi to produce such secreted peptides will thus help us to better understand their intimate biology and to prevent threats generated by mycotoxins on human health [12].

We recently documented the repertoire of secreted proteins (SPs) present in two arbuscular mycorrhizal fungi [13]. These fungi that belong to the Glomeromycotina subphylum, establish a unique type of symbiosis with land plants, allowing their host to get important benefits in terms of mineral and water acquisition, and of tolerance to various stresses including pathogens [14]. They are poorly characterized at the molecular level, since the first genome sequences of arbuscular mycorrhizal fungi were released only very recently [15, 16], and no stable genetic transformation methods are available for these species. Very recently, new genomes of Glomeromycotina species were released [17–19]. These fungi are considered as asexual fungi, even though some evidence suggests that mating may occur in some strains of *Rhizophagus irregularis* [20]. We identified numerous SPs of *Rhizophagus irregularis* that are predicted to be cleaved by KEX2. These proteins display a signal peptide and a repetition of nearly identical small motifs, all separated by a KEX2 cleavage site [13]. We thus wondered whether the number of these proteins in this early diverging fungus was in average similar to other fungal species and undertook a large-scale analysis.

To identify KEX2-targeted proteins that could produce secreted peptides, we downloaded protein catalogs from the DOE Joint Genome Institute and scanned them for the presence of i) a signal peptide, ii) internal repeats, and iii) KEX2 cleavage sites in-between repeats. With this pipeline, we analysed 250 protein catalogs representative of Ascomycota, Basidiomycota and early diverging fungi. We selected species for which lifestyle and morphology information were available. This study unveils within nearly all fungi the existence of a set of proteins that display a very typical sequence composition. We propose to describe these proteins as “KEPs” for *KEX2-processed repeat* *proteins.* Our work expands our current knowledge on fungal secreted peptides with the identification of new Ascomycota sexual pheromones, and new putative signalling or regulatory peptides in the entire fungal tree of life.

## Results

### Secretome analysis

**Fig. 1:**
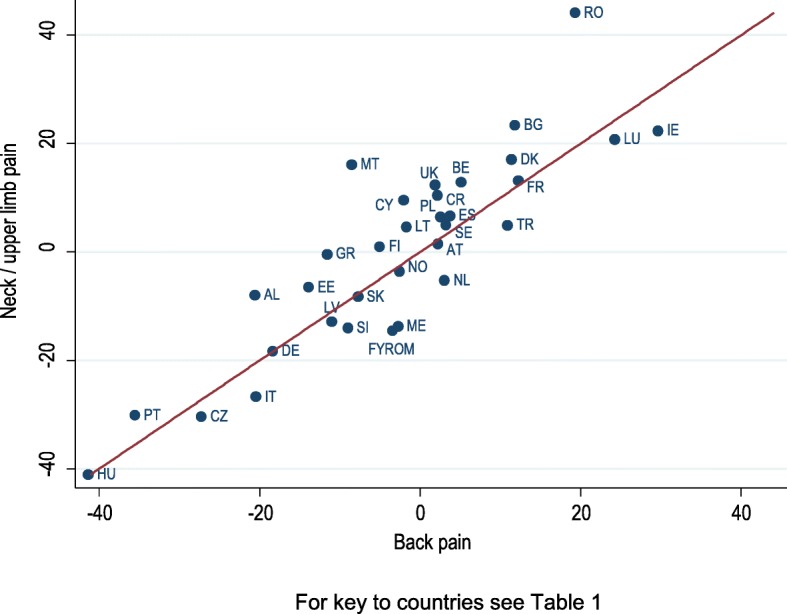
Proportion of secreted proteins (SPs) in 250 fungal species, with regard to fungal morphology (**a**), lifestyle (**b**) and lineage (**c**). U: yeasts, yeast-like and unicellular fungi, F: filamentous fungi, Endo: endophytes, Symb: symbionts, PatP: plant pathogens, PatA: animal pathogens, Sapr: saprotrophs, BasF: filamentous Basidiomycota, BasU: yeast, yeast-like and unicellular Basidiomycota, AscF: filamentous Ascomycota, AscU: yeast, yeast-like and unicellular Ascomycota, Chyt: Chytridiomycota, Muco: Mucoromycota, Zoop: Zoopagomycota, Micr: Microsporidia, Cryp: Cryptomycota. Statistical analysis was performed with one-way analysis of variance with post-hoc Tukey HSD test; **: *p* < 0.01). nd: fungal phylum for which statistical comparisons were not performed

**Fig. 2:**
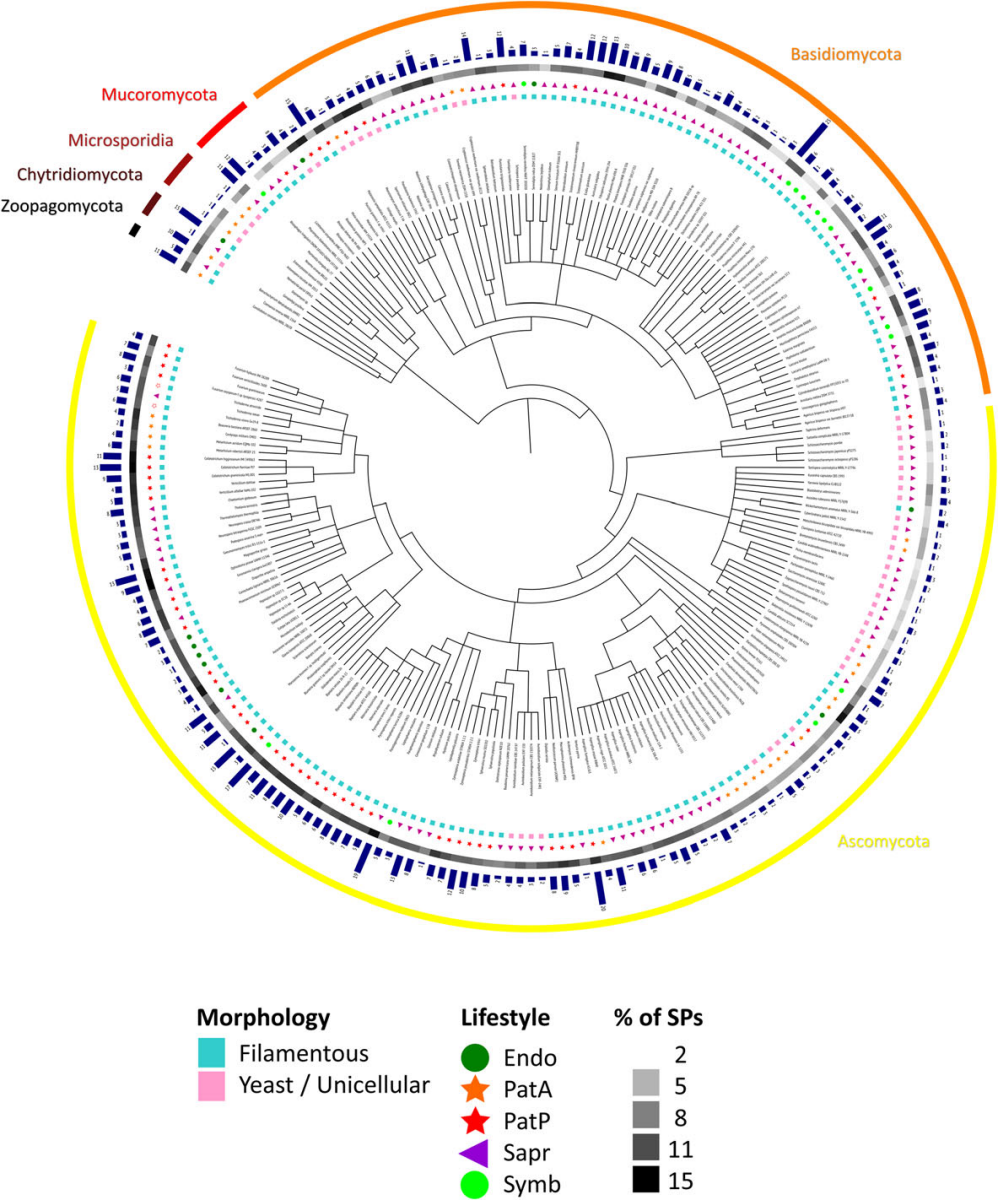
Overview of KEX2-processed repeat proteins (KEPs) occurrence with respect to fungal lineages. From the inside to the outside: fungal morphology, lifestyle, percentage of secreted proteins (SPs) in the proteome. Refer to legend for details. Dark blue bars heights reflect KEP numbers, also indicated on top of them. Mycoparasitic fungi are represented by red open stars. Endo: endophytes, Symb: symbionts, PatP: plant pathogens, PatA: animal pathogens, Sapr: saprotrophs. Full information is available in Additional fila 2 Table S2. A dynamic figure, with detailed information of the lineage, is available on https://itol.embl.de/, in the “Sharing data” section, with the iTOL login “freiditfrey”

With a survey of 250 secretomes, our study presents so far the most extended analysis of fungal secreted proteins. Before analysing KEX2-processed repeat proteins (KEPs), we compared the percentage of secreted proteins in each species, with respect to their lineage, their lifestyle or their morphology (Additional file 1 Table S1a). As shown in Fig. 1a, unicellular organisms display a smaller proportion of SPs than filamentous fungi. We also observe that plant pathogens display a larger secretome than animal pathogens, symbionts and saprotrophs (Fig. 1b). These observations fit with previous analyses [21]. For animal pathogens, it is important to note that they are represented by numerous yeast-like or intracellular organisms, known for their reduced genome size [22], while plant pathogens are almost all filamentous. Within Dikarya, yeast-like fungi from the Basidiomycota and filamentous fungi from the Ascomycota showed the larger proportion of SPs. The high proportion of plant pathogens in Ascomycota filamentous species can explain this observation. Yeast-like fungi from the Basidiomycota are however composed of fungi of very different lifestyles. Thus, their trend to invest a large proportion of their proteome in SPs is a unique feature that oppose them to yeasts from the Ascomycota (Fig. 1c). Within these secretomes, we then scanned for repeat proteins putatively cleaved by KEX2 (see Methods for details). In Fig. 2, we display the fungal species in which at least one KEP was observed, with information concerning their morphology and lifestyle (for details, refer to Additional file 1 Table S1a). In the Methods section, we specify the species in which proteases involved in KEP maturation were not found (Additional file 1 Table S1b) and we detail the 24 species that display no robust KEPs (Additional file 1 Table S1c and Table S1d). The dendrogram displayed in Fig. 2 is accessible as a dynamic tree on the iTOL website (see the legend of Fig. 2 for detailed explanations).

### Overview of KEX2-processed repeat proteins (KEPs)

**Fig. 3:**
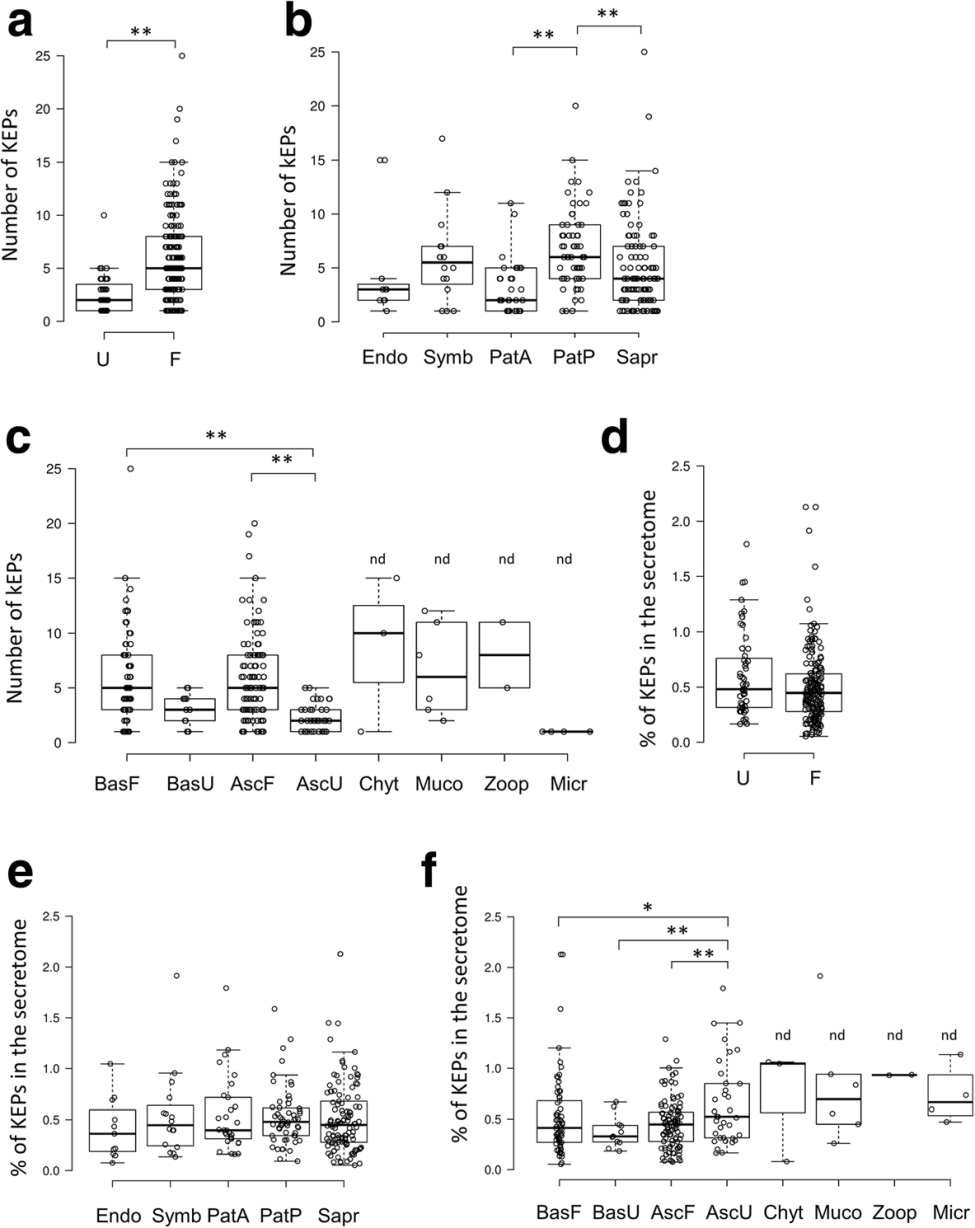
Distribution of KEX2-processed repeat proteins (KEPs) represented as absolute numbers (**a**-**c**) or relative to secretome size (**d**-**f**), with regard to fungal morphology (**a**, **c**), lifestyle (**b**, **e**) and lineage (**c**, **f**). U: yeasts, yeast-like and unicellular fungi, F: filamentous fungi, Endo: endophytes, Symb: symbionts, PatP: plant pathogens, PatA: animal pathogens, Sapr: saprotrophs, BasF: filamentous Basidiomycota, BasU: yeast, yeast-like and unicellular Basidiomycota, AscF: filamentous Ascomycota, AscU: yeast, yeast-like and unicellular Ascomycota, Chyt: Chytridiomycota, Muco: Mucoromycota, Zoop: Zoopagomycota, Micr: Microsporidia. Statistical analysis was performed with one-way analysis of variance with post-hoc Tukey HSD test; **: *p* < 0.01, *: *p* < 0.05. nd: fungal phylum for which statistical comparisons was not performed

We wondered whether the number of KEPs varied in the different fungal species. Filamentous fungi display a larger number of KEPs than unicellular fungi (Fig. 3a). Plant pathogens displayed a larger number of KEPs than animal pathogens or saprotrophs (Fig. 3b), and filamentous fungi from the Dikarya showed more KEPs than yeasts from the Saccharomycotina and Taphrinomycotina subphylums (Fig. 3c). Since expansions of KEPs could be easily explained by secretome size, we determined the percentage of KEPs in each secretome. This comparison revealed that morphology (Fig. 3d) or lifestyle (Fig. 3e) did not affect the proportion of KEPs. However, yeasts from the Saccharomycotina and Taphrinomycotina subphylums produced more KEPs relative to their secretome size (Fig. 3f). These proteins represent therefore a more important investment for these organisms, in comparison to other Dikarya. Too few species from the early diverging fungi were present in our study to compare them to Dikarya species. However, we could observe that some Chytridiomycota, Mucoromycota and Zoopagomycota species produce numbers of KEPs similar to Dikarya top-producing species.

**Fig. 4:**
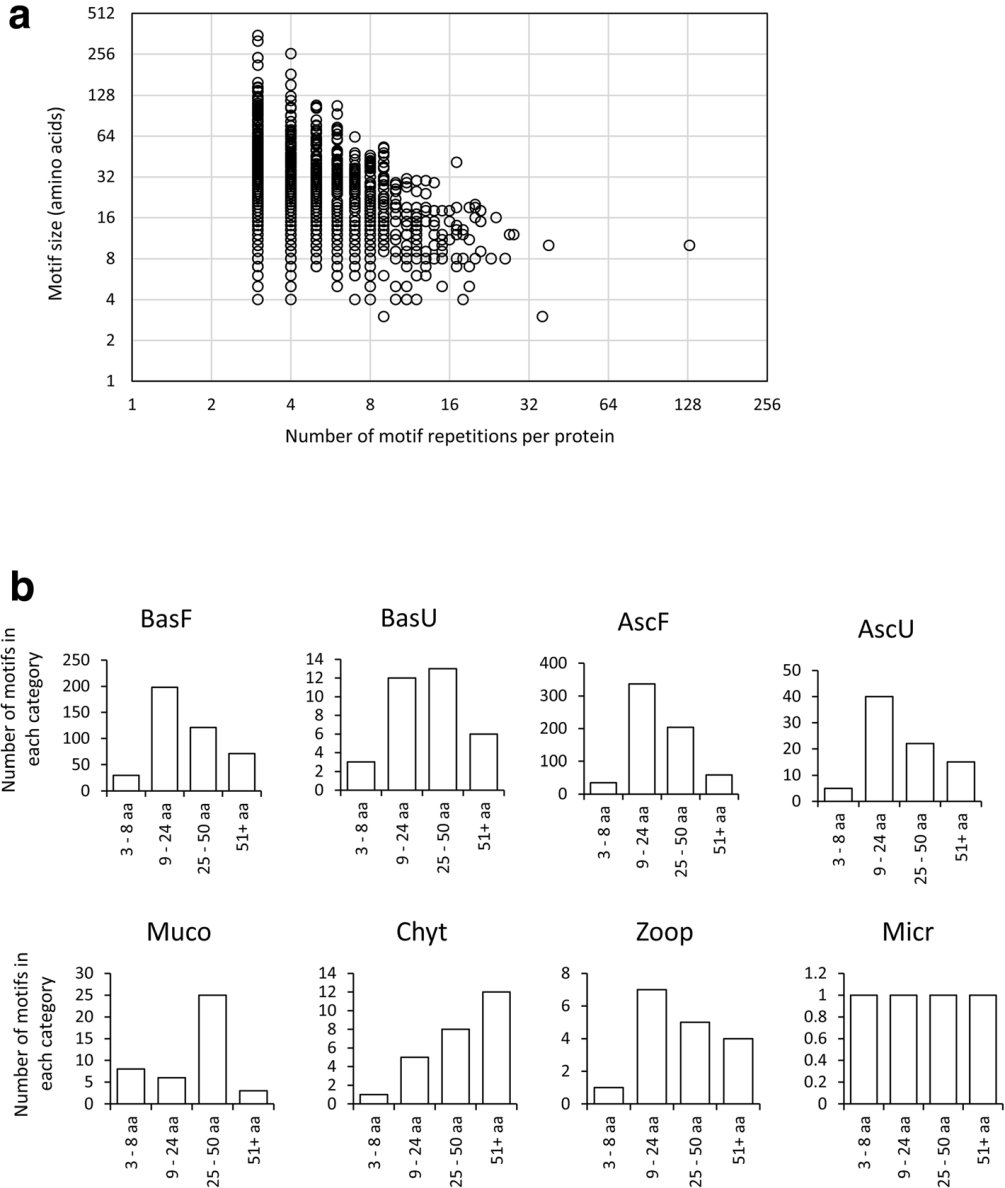
Overview of motifs identified in KEX2-processed repeat proteins (KEPs). **a** for each KEP, the amino acid length of the putative KEP-derived peptide is plotted against its number of repetitions. **b** Number of KEP-derived peptides, divided in four size classes, observed in the different fungal species. BasF: filamentous Basidiomycota, BasU: yeast, yeast-like and unicellular Basidiomycota, AscF: filamentous Ascomycota, AscU: yeast, yeast-like and unicellular Ascomycota, Chyt: Chytridiomycota, Muco: Mucoromycota, Zoop: Zoopagomycota, Micr: Microsporidia

We found that the repeated motifs of KEPs were also very variable in terms of size or number of repetitions. Their size ranged from three to 352 amino acids and the number of repetitions ranged from three (the minimal number allowed by the pipeline) to 129. This very high number of repetitions was an extreme case (found in a single protein of the plant pathogen *Melampsora lini*) since the second largest number of repetitions found was 38 (in *Rhytidhysteron rufulum*, Fig. 4a). We also noticed that the largest sizes of the repeated motifs were found in KEPs with low number of motif repetitions, and reciprocally the highest numbers of motif repetitions were found in KEPs with small size motifs (Fig. 4a). Based on known proteins falling into our definition of KEPs, we splitted the motif sizes into different classes. One class contains sizes comprised between nine and 24 aa. This size range corresponds to the known size distribution of Ascomycota α-type sexual pheromones [8]. We then counted the number of peptides in three other size categories: 3–8 aa, 25–50 aa and over 51 aa. In Dikarya, the 9–24 aa class was the most abundant except in yeast-like organisms from the Basidiomycota that produce longer putative peptides. Chytridiomycota and Mucoromycota also display peptides of larger sizes (Fig. 4b).

### Fungal species with large number of KEPs

**Fig. 5:**
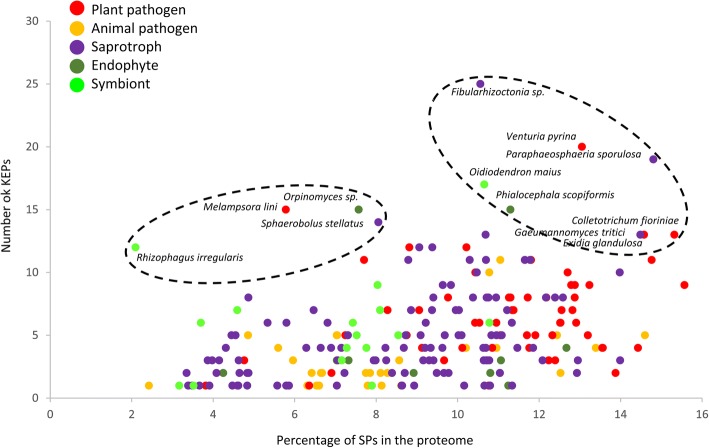
Number of KEX2-processed repeat proteins (KEPs) in each fungal species, with respect to the percentage of SPs in their secretome. Lifestyles are indicated with different color codes, as referred in the legend. Two groups of fungi, highlighted with a dotted-line circle display large numbers of KEPs. As indicated in the text, the left group is characterized by species with a large genome size

We then focused on species with large number of KEPs. To distinguish between species that differentially invest in SPs, we plotted the number of KEPs in function of the percentage of SPs for each species (Fig. 5 and Additional file 1 Table S1e). Twelve species focused our attention. Four fungi are characterized by a notable expansion of their genomes, all exceeding 100 Mbp: *Rhizophagus irregularis*, *Orpinomyces sp*. strain C1A*,* respectively a Mucoromycota and a Chytridiomycota, and two Basidiomycota: *Melampsora lini* and *Sphaerobolus stellatus.* These four fungi possess a relatively small percentage of SPs (Fig. 5)*.* The eight other fungi have a high percentage of SPs associated to a high number of KEPs. They are composed of six Ascomycota and two Basidiomycota. Altogether, these eight fungi are not characterized by a particular high genome size, with an average of 55 Mbp. These twelve fungi are all filamentous and interact with plants, either as plant biomass degraders (four fungi), plant pathogens (four fungi), symbionts (two fungi) or endophytes (two fungi), as indicated in Fig. 5. This observation suggests that KEPs may play a role in the diversity of mechanisms deployed by fungi to interact with plants.

Beyond the twelve above-mentioned species, we observed in the top-producing species several osmo- or xerotolerant organisms (*Wallemia ichthyophaga*, *Wallemia sebi*, *Zygosaccharomyces rouxii*, *Wickerhamomyces anomalus*) and many yeasts including four animal pathogens (Additional file 1 Table S1e). These yeasts conserve a high number of KEPs despite their reduced genome size, indicating important biological roles for KEPs in these species.

### Ascomycota sexual α pheromones

**Fig. 6:**
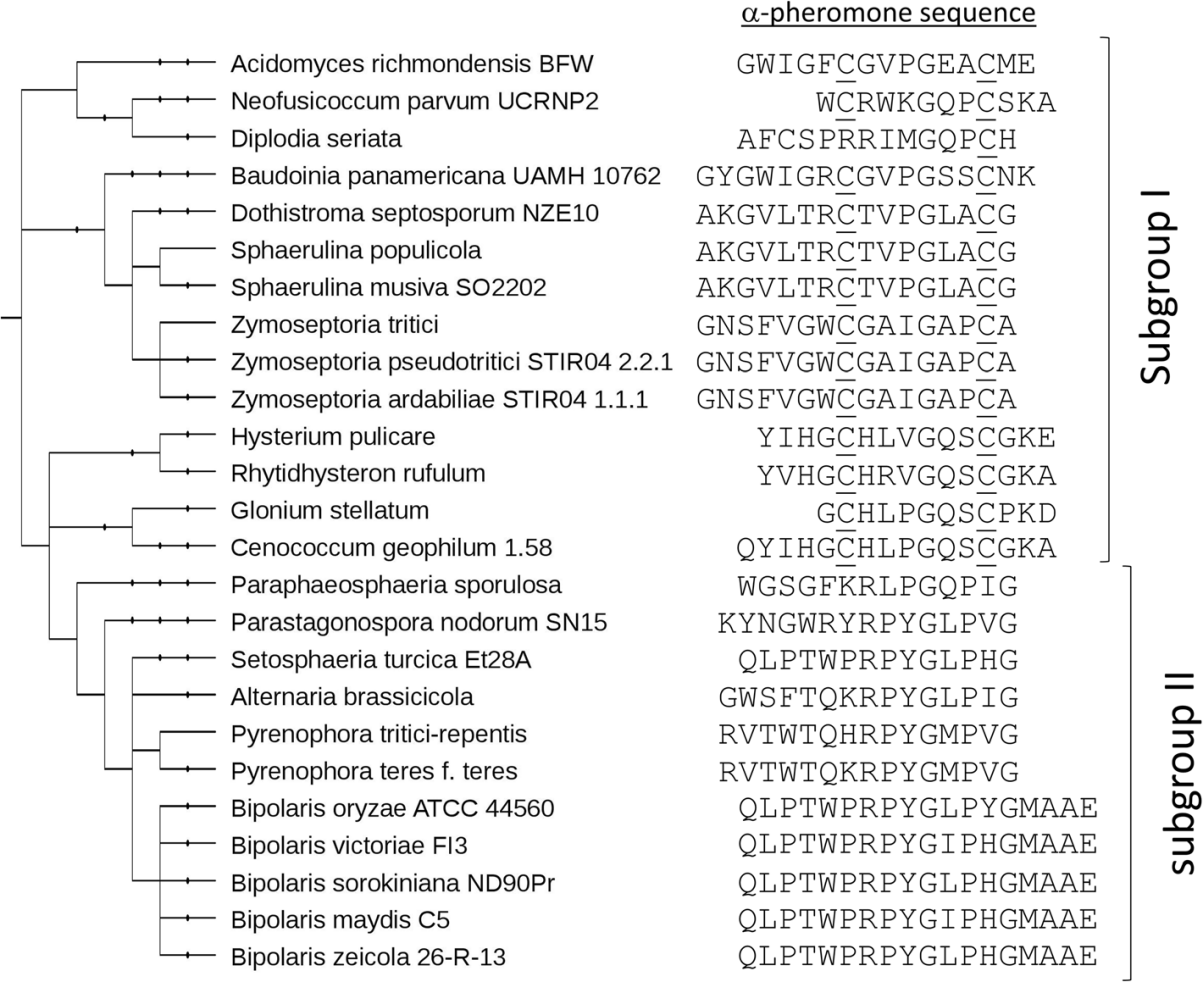
Alpha-type sexual pheromones identified in Dothidiomycetes. Two subgroups are observed: subgroup I with two conserved Cysteines (underlined) and subgroup II with the core motif PYGXPXG, unique to Pleosporales

Sexual pheromones observed in Ascomycota fall into two distinct categories. The “a-type” sexual pheromone is a post-translationally modified peptide. Its precursor protein does not enter the secretory pathway and possesses one copy of the peptide, located at the C-terminus. The protein is processed and matured in the cytoplasm and the generated peptide exits the cell via a transporter [23]. The second is the α sexual pheromone. It is produced from a long precursor that enters the secretory pathway and is processed by KEX1, KEX2 and STE13 proteases to finally release repeated peptides through exocytosis [23]. These peptides are not post-translationally modified. These latter proteins exactly fit our definition of KEPs. We therefore expected and indeed identified sequences corresponding to such proteins in Ascomycota secretomes. Through a BLAST-based search, Martin and co-workers reported an important number of Ascomycota α sexual pheromones [8]. Our pipeline expanded this number, and importantly, provided putative pheromone sequences for fungal subdivisions that have not been deeply explored so far. This was especially the case for Dothidiomycetes. In this subdivision, Martin et al. already showed a high diversity of sexual pheromone sequences among three fungal species: *Alternaria brassicicola*, *Phaeosphaeria nodorum* and *Pyrenophora tritici-repentis*. These three species have a α pheromone with a conserved PYG[L/M]P[I/V]G sequence at the C-terminus. We confirmed this observation for ten other dothidiomycete species, all belonging to Pleosporales (subgroup I, Fig. 6). However, 13 other dothidiomycete species displayed a very different α sexual pheromone with two conserved Cysteines, similarly to what is observed in Leotiomycetes and Sordariomycetes (subgroup II, Fig. 6, [8]). A similar putative sexual α pheromone was identified in the single xylonomycete species studied in our work, *Xylona heveae,* with the conserved RFCHLPGQGCSK motif (jgi|Xylhe1|266,977|gm1.2683_g, Additional file 2 Table S2).

As mentioned above, Ascomycota species produce a-type and α-type sexual pheromones. In Basidiomycota, sexual pheromones are only composed of a-type pheromones. Therefore, they do not use KEPs to produce mating signalling peptides. The STE13 signature (XA or XP following the KEX2-targeted dipeptide) is generally only dedicated to mature α-type sexual pheromones in Ascomycota. It is therefore not expected in basidiomycota KEPs. To our surprise, we identified a protein producing the putative repeated peptide QSWGGCGGRGTPCW in one Basidiomycota species – the yeast *Trichosporon oleaginosus* - (Additional file 2 Table S2). This peptide contains two conserved Cysteines, a N-terminal W (third position), and a C-terminal “PCW” motif that is for example well conserved in Fusarium species and other Sordariomycetes [8]. Furthermore, it contains conserved STE13 signatures following the KEX2 cleavage site. This amino acid composition thus presents striking and intriguing similarities with Ascomycota α sexual pheromone precursors.

### STE13 signature in KEPs

**Fig. 7:**
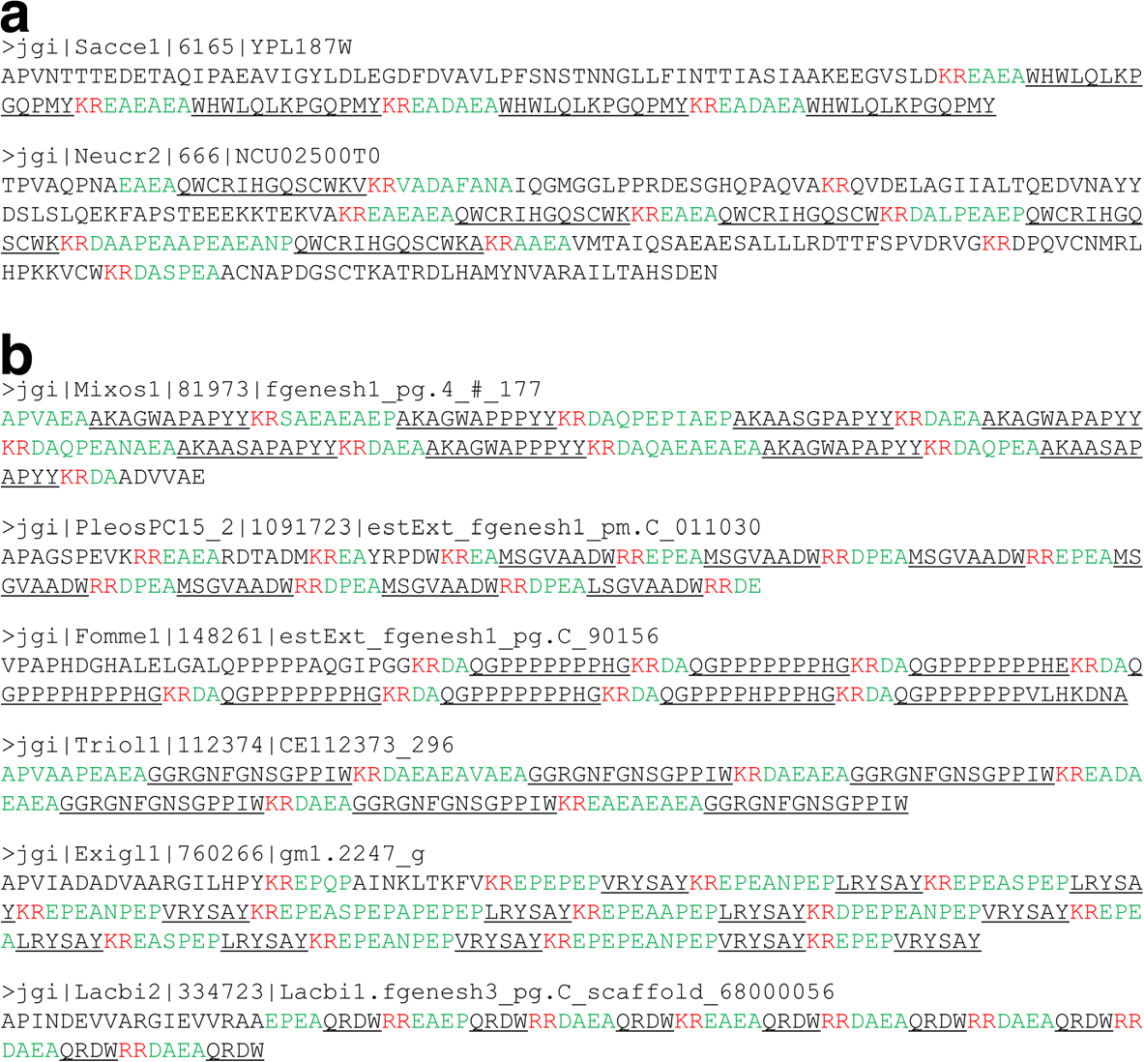
Example of sexual alpha pheromone in Ascomycota (**a**) compared to putative pheromones identified in Basidiomycota (**b**). KEX1/2 cleavage site is represented in red letters; STE13 cleavage site is represented in green letters; known (**a**) or deduced peptide sequence (**b**) is underlined

In Ascomycota, the KEX2 cleavage site present in the α sexual pheromone is followed by conserved amino acids removed by STE13 [8]. We therefore searched for this signature in all peptides obtained from our pipeline. While a STE13 cleavage site was expected for Ascomycota α sexual pheromones, it was not obviously predicted for other precursor genes, notably in Basidiomycota or in early diverging fungi. Indeed, Basidiomycota sexual pheromones are not composed of repeated peptides cleaved by KEX1/2 and STE13 proteases. However, in Basidiomycota, some KEPs displayed a very clear STE13 signature with a repetition of STE13-cleaved dipeptide, as is often the case in Ascomycota α sexual pheromones. Examples are given in Fig. 7, for species with diverse lifestyles. Two well-studied α sexual pheromone precursors from Ascomycota are depicted for comparison (Fig. 7a). The only distinction with Ascomycota α sexual pheromones is the sequence of the released peptide, but the protein structure is here perfectly canonical (Fig. 7b). Other clear STE13 signatures were found in KEPs from the Glomeromycotina *Rhizophagus irregularis* (Additional file 2 Table S2). This surprising observation reveals that the protein composition present in α sexual pheromones has been maintained in Basidiomycota species for other purposes than sexual reproduction. For Mucoromycotina and other early diverging fungal species, the role of these KEPs remains to be elucidated.

### Conserved secreted peptides across different species

**Fig. 8:**
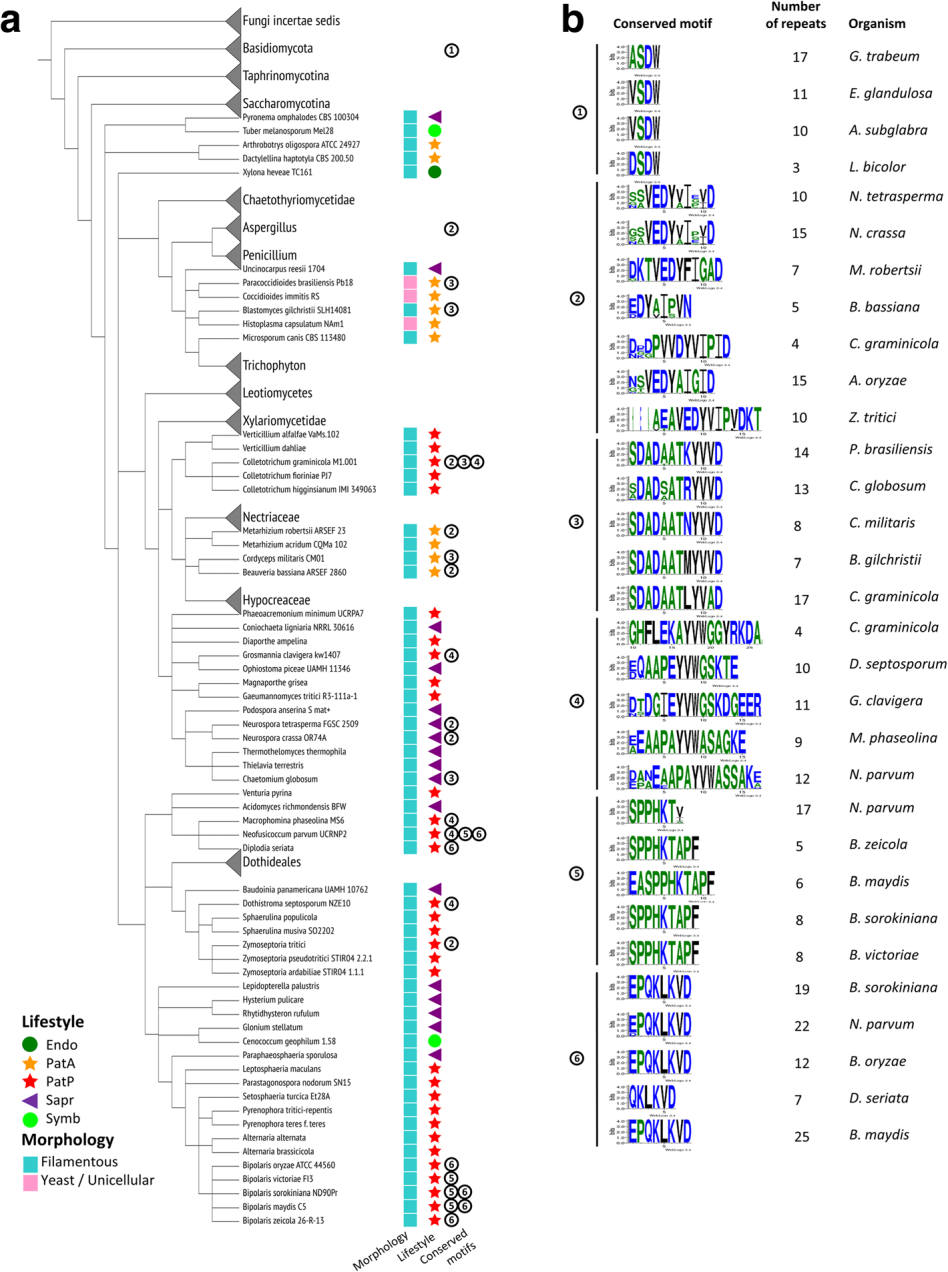
Conserved peptides identified throughout the pipeline. **a** Selection of fungal species displaying conserved motifs. Endo: endophytes, Symb: symbionts, PatP: plant pathogens, PatA: animal pathogens, Sapr: saprotrophs. Categories of conserved motifs are depicted by encircled numbers. Categories are depicted in (**b**). They correspond to groups of repeated motifs with a similar sequence, identified in at least four species. Similarity was determined by manual curation. For each fungal species, the conserved motif and its number of repetition is indicated. Motif 4 in *C. graminicola* was cropped to highlight its conserved sequence. Branches of the tree where collapsed when species displayed no conserved peptide. In the case of Basidiomycota and Aspergillus groups, branches were collapsed for space saving (only some species of these groups indeed contain a conserved peptide). Refer to Additional file 2 Table S2 for detailed information

We then analysed through manual curation the diversity of peptide sequences obtained with our pipeline. Peptides that were only conserved at the genus level were not taken into account. Moreover, only conserved peptides identified in at least four different species were considered. The sexual pheromones represented the major group of conserved peptides within Ascomycota, as expected. We also expected precursors of toxic cyclic peptides found in Ascomycota: ustiloxins [11] and phomopsins [5]. Aspergillus genomes were explored in detail by Nagano et al. in 2016 in order to identify new precursor genes resembling ustiloxin precursors (called ust-RiPS). These genes are present in a conserved genomic context that facilitates their identification, with ustYa/Yb and other conserved genes. Almost all precursor proteins encoding ribosomal peptides described by Nagano et al. (2016) display KEX2 cleavage sites in-between repeated peptides. Our pipeline allowed the identification of the same precursor proteins. Ustiloxins and phomopsins display a conserved EDYXI core sequence in their repeated peptides (motif 2, Fig. 8). We further confirm that they are present in a wide range of pathogenic and saprotrophic species, as reported by Ding and coworkers (*Zymoseptoria tritici*, *Colletotrichum graminicola*, *Metarhizium robertsii*, *Beauveria bassiane*, *Neurospora tetrasperma* and *N. crassa* [5]). The same work on Aspergillus genomes, taking into account the vicinity of ustYa/Yb genes, reported peptide precursor genes with very different sequences to ustiloxin precursors [11]. We further extended this analysis. For example, we observed the ust-RiPS type-1a [11] in *Dothistroma septosporum* and *Fusarium graminearum* (Additional file 2 Table S2).

Apart from these well described peptide precursors, we also identified in Pezizomycotina new conserved KEPs predicted to release peptides with the following core sequence: DADAATXYV[A/V]D, YVW[G/A][G/S], SPPHKT and QKLKVD (motifs 3 to 6, Fig. 8). Many of these conserved peptides were present in pathogenic species (motif 1, Fig. 8). We also identified a conserved XSDW repeated motif in Basidiomycota, present in an ectomycorrhizal fungus and in saprotrophic species (Fig. 8).

### Expression of KEP-coding genes

Our initial work on the arbuscular mycorrhizal fungus *R. irregularis* allowed the identification of seven KEPs with differential expressions [13]. We thus searched for gene expression datasets in the literature to more largely confirm the importance of KEPs. In fourteen other fungi (two saprophytes, two animal pathogens, one plant endophyte and nine plant pathogens, Additional file 3 Table S3), when genes were present in the studies, we systematically observed expression values for the identified KEP-coding genes. When differential expression was available (from samples obtained with different growth stages or conditions), we identified 25 out 56 KEPs either down- or up-regulated (Additional file 3 Table S3). For example, in time course experiments involving the plant pathogens *Magnaporthe grisea* and *Ustilago maydis* with their host [24, 25], six out of nine and four out of five KEPs were respectively differentially expressed. This observation further supports a decisive role for KEPs in fungal biology, notably during plant-fungus interactions.

## Discussion

Our work presents the largest analysis of fungal secreted proteins predicted to release repeated peptides through KEX2 activity. The best-characterized representatives of these proteins are the α sexual pheromones of Ascomycota. In these fungi, sexual pheromones are of two types. The “a” pheromone is produced from a single peptide located at the C-terminus of a precursor protein and requires post-translational modifications for activity. The “α” pheromone is different. It is produced from a precursor protein that enters the secretory pathway and presents repetitions of nearly identical peptides that are produced by the concerted action of KEX1, KEX2 and STE13 proteases. The released peptides are not post-translationally modified and exit the cell through exocytosis. We hypothesized that many proteins with characteristics similar to α sexual pheromones would be present in many if not all fungal species. The rationale was that a few species, scattered all along the fungal kingdom, were shown to possess such proteins [5, 10, 11, 13, 26]. To make a comprehensive analysis, we performed a de novo secretome prediction from 250 fungal protein catalogs.

Our analysis confirmed previous studies on fungal secretomes [21, 27]. Unicellular Ascomycota, mostly represented by Saccharomycotina and Taphrinomycotina subphylums, have the lowest content of secreted proteins in the Dikarya. Furthermore, we observed a higher proportion of SPs in plant pathogenic species in comparison to the other studied lifestyles, and we did not see a difference between animal pathogen and saprotrophs, similarly to previous analyses [21]. However, the distinction that we provide in Basidiomycota between filamentous and yeasts species unravels interesting differences not referenced so far. Indeed, yeasts in this subphylum display a greater proportion of SPs than their filamentous counterparts. This is in sharp contrast to what was observed in Ascomycota. We also observed that these yeasts from the Basidiomycota tend to produce longer peptides through KEP processing than the other Dikarya, indicating that they differently evolved the use of these peculiar proteins. Also, filamentous fungi from the Ascomycota display a greater percentage of SPs than filamentous species from the Basidiomycota. The high proportion of plant pathogen species in filamentous Ascomycota can explain this difference. In our dataset, among 56 plant pathogens, 45 are filamentous Ascomycota. Besides, the observation that Chytridiomycota have a greater amount of SPs than Mucoromycotina [27] is also supported by our data.

Our pipeline identified several species that displayed a large number of KEPs, both in absolute numbers and relative to their secretome size. As already mentioned these species interact with plants or tolerate difficult environments. Several yeasts are also in this category, including animal pathogens. For pathogenic species, or endophytes and symbionts, it is tempting to speculate that KEPs may produce secreted peptides interfering with host defence, in order to promote colonization. We indeed observed several KEP-coding genes induced during interaction. Secreted peptides may also structure hyphal surface [9] or damage host cells [28]. For osmo- and xerotolerant species producing a large number of KEPs, we can speculate that KEP-derived secreted peptides have a structural role in the fungal cell wall, similar to *U. maydis* Rep1 protein [9]. Such cell wall modifications may allow to grow in extreme conditions.

### Enrichment of the catalogue of α-type pheromones in Ascomycota

To describe the richness of peptides involved in sexual reproduction, a detailed analysis of 69 Ascomycota species was performed by Martin and coworkers [8]. It highlighted the diversity of α sexual pheromones. We completed this study and further expanded our knowledge on species that had not been explored so far. Interestingly, we observed that Dothidiomycetes present a larger diversity in peptide sequences than previously known. Indeed, we identified two different subgroups displaying clearly different peptide sequences. Pleosporales form a monophyletic group with a conserved PYG[L/M]P[I/V]G sequence, while all other Dothidiomycetes display a peptide sequence that was more similar to Leotiomycetes and Sordariomycetes peptides. Our study also presents for the first time a candidate sexual α pheromone in a Xylonomycete species, *Xylona heveae*. This discovery will help to study its reproductive mode. Overall, the identification of these sexual pheromones will also be useful to study or trigger mating programs in fungi that are sexually recalcitrant.

### Conservation of sexual pheromones in “asexual” fungi

Numerous works have pointed out the existence of a putatively efficient sexual machinery in fungi that always behaved as asexuals. Thus, it was suggested that the number of true asexual fungi was overestimated [29]. It is indeed surprising that genes involved in sexual reproduction are still conserved in many species described as asexuals. Sexual pheromone genes are for example present in the following presumed asexual fungi: *Cladosporium fulvum*, *Aspergillus niger*, *Verticillium dahlia*, *Fusarium oxysporum* and *Trichoderma reesei* (this study, [8]). Either these fungi are indeed true asexual and have conserved their α-type pheromone gene for a novel function, or they are anamorph-species with no known teleomorph, or else they possess a cryptic sexual or parasexual cycles that require special conditions to be observed. It is the case for many *Aspergillus* species in which specific laboratory conditions are necessary to trigger sex ([30] and references therein). It is also important to consider that sexual pheromones have other important sex-independent roles such as biofilm production or conglutination of hyphae ([29] and references therein).

### Discovery of KEPs with α-type pheromone signatures in Basidiomycota

While Basidiomycota do not use α-type pheromones for their sexual reproduction, we identified several proteins with α-type pheromone features in their secretomes. These KEPs present a clear STE13 signature, identical to that of α-type pheromone protein precursors of Ascomycota. Given the presence of these proteins in Basidiomycota, it is therefore tempting to speculate that cognate G-Protein Coupled Receptors (GPCRs) are also present in these fungi to perceive these peptides. GPCR are involved in the perception of sexual pheromones in Dikarya, with for example STE2 and STE3 proteins respectively involved in the perception of the a- and the α-type pheromones in yeast. We thus speculate that KEPs with clear STE13 signatures may act as hormones or pheromones regulating distinct cellular programs. Several cellular processes require complex integration of endogenous signals or need to cope with external cues: hyphal polar growth, septation, branching, fusion or healing, hyphal network coordination and production of sexual and/or vegetative reproduction bodies and spores. KEPs may be precursors of peptides regulating some of these processes.

Within Basidiomycota, our pipeline also identified a KEP in the yeast *Trichosporon oleaginosus* with striking peptide sequence similarities with Sordaryomycetes α-type pheromones. However, scrutiny of *T. oleaginosus* genome assembly allowed the identification of a putative a-type pheromone gene [31]. This observation suggests that the Ascomycota-like sexual pheromone peptide that we identified in this species may have another function than sexual reproduction in *T. oleaginosus*.

The STE13 signature is not present in the KEX2-processed precursor protein Rep1 of *U. maydis* that produces peptides attached to fungal hyphae [32]. It is also absent in Ascomycota proteins releasing toxic cyclic tetrapeptides [5, 11] or in precursors of epichloëcyclins [10]. KEPs displaying peptides with a STE13 signature are therefore good candidates for the release of peptidic hormones that may have distinct roles from sexuality and may regulate other aspects of fungal biology. In the future, it will be interesting to determine which GPCRs are involved in the self-perception of these molecules. A non-exclusive hypothesis would be that these secreted peptides are also involved in the interaction with other living organisms, thus acting as effectors.

### Ustiloxins, phomopsins and other post-translationally modified peptides

As mentioned above, cyclic peptides are produced in Ascomycota from KEX2-processed precursor proteins. Initially these peptides were mistakenly thought to be synthesized through a non-ribosomal pathway. In fact, they belong to the family of Ribosomally synthesized and post-translationally modified peptides (RiPPs), and they act as mycotoxins. The best described are ustiloxins [33] and phomopsins [5]. Similarly to Ascomycota α pheromone precursor proteins, they display a signal peptide and a repetition of peptides all separated by KEX2 cleavage sites. However, STE13 is not predicted to process the initial protein. Other proteases, involved in the maturation of the precursor, were identified in close chromosomal vicinity of the RiPP genes. Additional genes are described in these genomic clusters and are good candidates for the activation, maturation and release of the peptides [5, 11]. Eventually, their maturation leads to cyclization. A conserved Tyrosine is essential for this structural modification. It will be important to determine whether KEPs containing a conserved Tyrosine also undergo cyclization. Besides phomopsins and ustiloxins, extensive analyses of Aspergillus genomes allowed the identification of other putative cyclic compounds [11]. Based on the similarity of sequences between known ribosomally synthesized mycotoxins and the extended list of KEPs that we describe in this work, it will now be easier to predict the putative presence of mycotoxins in a wide variety of fungal species. This resource is of prime interest with regard to food safety threats generated by mycotoxins [12].

### Identification of new conserved secreted peptides

Several species of different genera or even classes displayed similar peptides. Such species conservation was already observed with phomospins [5]. In our case, when a conserved motif was found in different species, what generally differed between species was the number of repetitions of the conserved peptide. In most of these KEPs, the conserved peptide belonged only to pathogenic species, thus suggesting a possible role in infection processes. It is indeed possible that some of these peptides act as effectors and target host defence mechanisms. In *Candida albicans*, a peptide released after KEX1 and KEX2 cleavage acts as a toxin to target human epithelial membranes [34, 35]. The protein involved in this production displays a repetition of non-identical peptides, and only one has a virulence function. As discussed before, peptides derived from KEPs identified in this work are thus good candidates for interference with host biology. Another possibility would be that KEP-derived peptides regulate a fungal signalling pathway triggering virulence programs. This was recently observed in *C. neoformans* that perceives a self-produced 11 amino acid long peptide promoting its virulence [28]. Interestingly, this peptide was recently shown to activate *C. neoformans* sexual programs [36].

We identified a new conserved peptide in several pathogenic species and one saprotroph, *Chaetomium globosum*. Recently, a report suggested that this fungus was involved in a case of leaf spot disease [37] and onychomycosis [38]. The conservation of pathogenic KEPs may therefore allow to fungi of different lifestyles to develop opportunistic pathogenic behaviours.

### KEX2-processed repeat proteins in early diverging fungi

Members of the Mucoromycotina and the Blastocladiomycota, respectively, use apocarotenoids and sesquiterpenes as sexual pheromones (for review, [39]). The chemical nature of pheromones in the other early diverging fungi is largely unknown. Our data reveal that KEPs are present in these fungi. The role they possibly play in their sexuality or in other biological processes will have to be determined. The arbuscular mycorrhizal fungus *Rhizophagus irregularis* presents a high number of KEPs while it has the lowest percentage of SPs throughout all fungal species studied in this work. Furthermore, most of its KEPs display STE13 signatures after the KEX2 cleavage sites. These signatures are nearly identical to those of *S. cerevisiae* canonical sequences. This observation is surprising given that *R. irregularis* has diverged very early from Saccharomycotina. Moreover, it is the unique early diverging fungus that displays such features. *R. irregularis* has long been described as an asexual fungus, but recent evidence suggests that it may have a cryptic sexuality [20, 40]. This fungus also displays an expansion of KEPs and the deduced peptides are intriguingly rich in Tyrosines. Further investigations will be important to determine whether these peptides undergo cyclisation, as it is the case in RiPPs [5, 11].

### Origin of KEX2-processed repeat proteins

We provide evidence that KEPs are present in nearly all fungi explored in this study. Their structure resembles that of precursors of neuropeptides or other peptide hormones found in animals. In animals, proteins presenting repeated peptides are also cleaved at KR, RR or KK sites (Rholam and Fahy 2009). The cleavage is performed by proteases of the KEX2 family, named proprotein convertases. KEPs therefore seem to be present at the base of the opisthokont group. Besides, our pipeline does not identify good KEP candidates in plants (data not shown), most certainly because they do not contain KEX2-like proteins [41].

## Conclusion

In this work, we compile a list of 250 publicly available fungi and provide information on their lifestyle and their morphology. Our large-scale secretome analysis highlighted that yeasts from the Basidiomycota display a much larger percentage of secreted proteins than filamentous fungi of the same phylum, in clear opposition with Ascomycota. Through the survey of KEX2-processed repeat proteins, we identified a catalogue of novel putative secreted peptides in fungi. Some of these peptides could correspond to yet unknown sexual pheromones, mycotoxins, while others may act as virulence factors. We also show evidence that Basidiomycota and Glomeromycotina have conserved proteins with striking similarities to α-sexual pheromones so far only described in Ascomycota. The peptides derived from these proteins may play signalling roles unsuspected so far, indicative of a neofunctionalization of this type of proteins with a very ancient origin. Overall, our study opens interesting avenues for further work to discover new hormones (sexual or non sexual), mycotoxins and effectors in fungi.

## Methods

### Data description and classification

Publicly available protein catalogs of 250 fungal species were retrieved from the JGI database. Eighty-four, 145, and 21 datasets corresponded respectively to Basidiomycota, Ascomycota and early diverging fungi. Lifestyles were sorted so that 11, 94, 127 and 18 fungi were respectively classified as endophytes, pathogens, saprotrophs and beneficial symbionts. We also separated filamentous fungi (182 species) from unicellular, yeast or yeast-like fungi (68 species). The information concerning the 250 species, together with publication records for each genome, is displayed in Additional file 1 Table S1a.

### Search for KEX1, KEX2 and STE13 conservation

We performed a BLAST analysis to search for the presence of KEX2 but also KEX1 and STE13, two other proteases involved in the maturation of Ascomycota α-type pheromones. While KEX2 cleaves at the C-terminus of the KR/RR/KK dipeptide, KEX1 cleaves at its N-terminus. STE13 removes additional dipeptides following the cleavage site of KEX2 with a preference for XP or XA dipeptides. We used *Saccharomyces cerevisiae* protein sequences (KEX1, YGL203C; KEX2, YNL238W; STE13, YOR219C) as queries in a BLASTP analysis against NCBI refSeq_protein database, with a threshold e-value of 0.001. Only 15 available protein catalogs displayed a lack of at least one of these proteins (Additional file 1 Table S1b). KEX2 was for example absent in Microsporidia species, in two Taphrinomycotina, two Agaricomycotina and one Ustilaginomycotina species. Microsporidia are unique since they are the only species were the three proteases are absent. We kept anyway all the fungal protein catalogs for further analysis since other proteases could processed our proteins of interest in these 15 genomes. Moreover, we could not exclude that the absence of these proteases was simply due to incomplete genomic data.

### Pipeline description

The following pipeline is illustrated in Additional file 4 Figure S1 and the script can be downloaded from the link “Download repeatSearch” in Additional file 2 Table S2 (see Additional file 2 Table S2 legend for detail).

After protein catalog retrieval from the JGI, proteins present in exact duplicates were removed. SignalP4.0 [42] was used first and unselected proteins were then scanned with SignalP3.0 [43]. Proteins selected by either software were retained. We then discarded membrane-located proteins using TMHMM [44]. SignalP3.0, SignalP4.0 and TMHMM were used with default parameters. Protein sequences deleted of their signal peptide were then cleaved in silico at every KR or RR site. Proteins giving at least four fragments of at least two amino acids were selected. The other proteins were cleaved in silico at every KK site to conserve proteins giving at least four fragments of at least two amino acids. The C-terminal part of the protein was systematically excluded from our analysis since it produced in many cases a long fragment that distorted subsequent alignments and biased the average size of the repeated motif (motifs present in this part of the protein were recovered afterwards, see below). For each protein of interest, fragments were then sorted from the smallest to the largest. The smallest fragment was converted to a motif using the iupac2meme tool from the MEME suite [45], with default parameters. With Fimo [46], we used this motif to determine if the other fragments of the proteins presented sequence similarity. This search was performed with default parameters. All similar fragments selected by Fimo were then sorted apart to create a pattern. Fragments that did not match with the query were then again sorted from the smallest to the longest to perform a new run of iupac2meme conversion and Fimo analysis. This procedure was repeated until all fragments were integrated into a pattern or excluded. Patterns corresponding to at least three fragments were then conserved. The corresponding fragments were aligned with clustalw2 [47] and this alignment was used to create a logo with Weblogo [48]. We set the minimal number of repeats to three because in preliminary optimizations, this greatly reduced the number of false positives. In addition, most KEPs described in the literature have at least four repeats [8]. This is not the case for some precursors of α sexual pheromones, but these proteins were already well documented in a survey of Ascomycota sexual pheromones [8].

As mentioned above, the C-terminal fragment of the protein (after the last KR/KK/RR site) was excluded from the initial analysis. In order to recover a repeated motif that could be present in this part of the protein, we searched with Fimo if previously identified motifs in the protein were present in this protein fragment. This strategy helped for example to recover the fourth motif present at the C-terminal part of the *S. cerevisiae* MFalpha1 protein (YPL187W). The “Mydomains” tool of Prosite (https://prosite.expasy.org/mydomains/) was then used to draw a picture of each proteins, where the repeated motifs start and end at the correct amino acid positions (Additional file 2 Table S2).

Eleven protein catalogs did not present any proteins with at least three repeated motifs interspaced by KEX2-cleavage sites and were discarded (Additional file 1 Table S1c). This can be easily explained for *Pneumocystis jirovecii*, *Encephalitozoon cuniculi* and *Encephalitozoon romaleae* for which we were unable to find a KEX2 protein through BLAST analysis. Besides, we cannot exclude that KEX2 in these fungi cleave to unusual sites that are not KR, RR or KK.

We then removed proteins for which the total size occupied by the repeated motifs represented less than 15% of the protein length. For this, we multiplied the size of the minimal repeated motif by the number of repetitions in the protein and divided by the length of the protein. Below a 15% threshold, we generally observed long proteins with motifs separated by variable regions. This feature does not fit the typical structure of documented KEPs, where repeated motifs are compacted in clusters in one region of the protein. Thirteen protein catalogs did not meet the 15% threshold and were discarded (Additional file 1 Table S1d). Too few mycoparasytic fungi were present in this analysis. We only display information relative to their KEPs in Fig. 1a, Fig. 1c, Fig. 2, Additional file 1 Table S1 and Additional file 2 Table S2 but do not include them in other analyses. We ended up with 226 species (Fig. 2) that produced a total of 1193 KEPs (Additional file 2 Table S2). Within these KEPs, two and 77 produced respectively three and two different types of repeated motifs within their sequence. All the other KEPs (1114 proteins) produce one type of repeated motif (Additional file 2 Table S2).

The amino acids present after the KEX2 cleavage were recorded in order to identify STE13 signatures (XA or XP). They are displayed in Additional file 2 Table S2. Analysis through manual curation of each KEP for which at least one XA or XP dipeptide was present allowed the identification of STE13 signature for which examples are provided in Fig. 7.

### Statistical analyses

The normal distribution of data was analysed with a Shapiro test (Pval < 0.01) and an ANOVA was performed, followed by a Tukey post-hoc test to compare all pairs of means.

## Additional files

Additional file 1: **Table S1.** Overall description of the species identified in the pipeline. (XLSX 65 kb)

Additional file 2: **Table S2.** Full description of the different KEPs identified in the pipeline. (PDF 260 kb)

Additional file 3: **Table S3.** Expression analysis of a selection of KEP-coding genes. (XLSX 54 kb)

Additional file 4: **Figure S1.** Scheme depicting the informatic pipeline used to identify KEX2 processed repeat proteins (KEPs). (PDF 212 kb)

## Abbreviations

Mbp: Million base pairs
A: Alanine
R: Arginine
N: Asparagine
D: Aspartate
C: Cysteine
E: Glutamate
Q: Glutamine
G: Glycine
H: Histidine
I: Isoleucine
L: Leucine
K: Lysine
M: Methionine
F: Phenylalanine
P: Proline
S: Serine
T: Threonine
W: Tryptophane
Y: Tyrosine
V: Valine
