# Correction to: Identification of new signalling peptides through a genome-wide survey of 250 fungal secretomes

**DOI:** 10.1186/s12864-019-5466-y

**Published:** 2019-01-30

**Authors:** Morgane Le Marquer, Hélène San Clemente, Christophe Roux, Bruno Savelli, Nicolas Frei dit Frey

**Affiliations:** Laboratoire de Recherche en Sciences Végétales, CNRS, UPS, Université de Toulouse, 24 chemin de Borde Rouge, Auzeville, BP42617, 31326 Castanet Tolosan, France

**Correction to: BMC Genomics**

**https://doi.org/10.1186/s12864-018-5414-2**

Following the publication of this article [1] the authors noted that the image in Fig. 1 was incorrect. Due to a typesetting error an incorrect image was processed as Fig. 1, and the publisher apologizes to the authors and readers for the inconvenience. The correct figure is reproduced in this erratum:

**Fig. 1:**
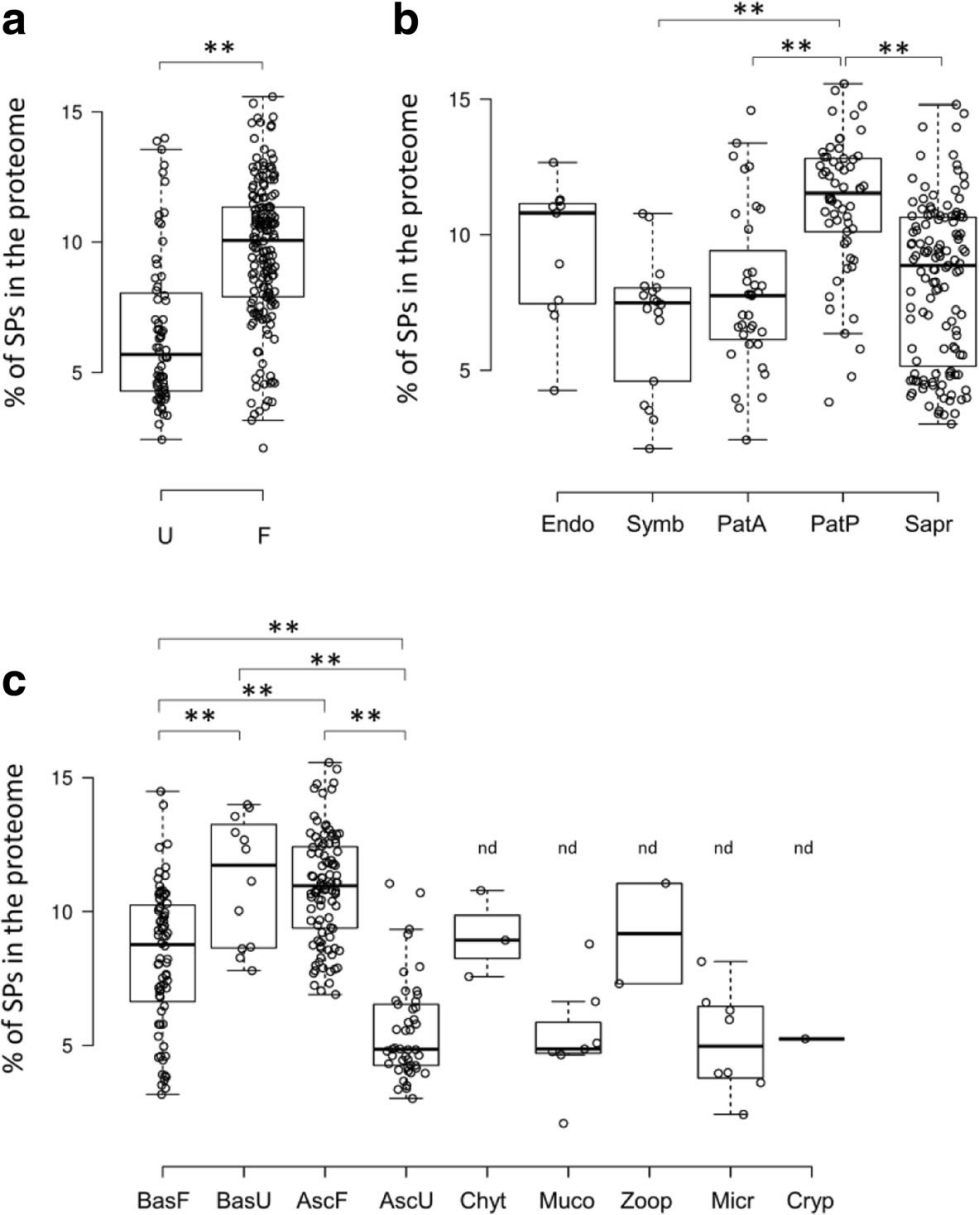
Proportion of secreted proteins (SPs) in 250 fungal species, with regard to fungal morphology (**a**), lifestyle (**b**) and lineage (**c**). U: yeasts, yeast-like and unicellular fungi, F: filamentous fungi, Endo: endophytes, Symb: symbionts, PatP: plant pathogens, PatA: animal pathogens, Sapr: saprotrophs, BasF: filamentous Basidiomycota, BasU: yeast, yeast-like and unicellular Basidiomycota, AscF: filamentous Ascomycota, AscU: yeast, yeast-like and unicellular Ascomycota, Chyt: Chytridiomycota, Muco: Mucoromycota, Zoop: Zoopagomycota, Micr: Microsporidia, Cryp: Cryptomycota. Statistical analysis was performed with one-way analysis of variance with post-hoc Tukey HSD test; **: *p* < 0.01). nd: fungal phylum for which statistical comparisons were not performed

The original article has been corrected.
