# Supplementary material for: Identification of new signalling peptides through a genome-wide survey of 250 fungal secretomes
Source: BMC Genomics. 2019 Jan 18;20:64. doi: 10.1186/s12864-018-5414-2 (PMC6339444; doi:10.1186/s12864-018-5414-2)
Supplement: Supplementary file 2 — Table S2. Full description of the different KEPs identified in the pipeline. (PDF 260 kb) [file 12864_2018_5414_MOESM2_ESM.pdf]

Table S2: Full description of the different KEPs identified in the pipeline.

Data is available here:
http://www.polebio.lrsv.ups-tlse.fr/RhizoCleavedwithcterDomain/

In the table, from left to right: Scientific Name (NCBI server, May 2018), Taxonomy ID (NCBI), Protein Id (protein sequence is available on click; the Signal Peptide is removed), Weblogo of the motif identified in the KEP (motif alignment is available on click), Picture of the protein (each colored circle corresponds to a repeated motif with the exact position of the motifs along the protein), Size of the protein, Number of types of repeated motifs, Number of motif repetitions, Software that identified the Signal Peptide (note that first SignalP4.0 (SP4) was used and in all remaining proteins a SignalP3.0 (SP3) was performed; thus "SP4" also contains proteins that could have been selected by SignalP3.0, Length of the motif, P1 to P4: identity of the amino acids present after the KEX2 cleavage site (all records from the different motifs are pooled in the same column; P1: first position, P2: second position, P3: third position, P4: fourth position after the KEX2 cleavage site), nature of the cleavage site (either KR/RR sites or KK sites), conserved positions based on the motif alignment.

Please note that the ***Protein Id*** and the ***Logo*** are clickable links that open full informations on the protein sequence and the alignments of the motifs, respectively.
The "Download repeatSearch" link (top left corner) allows the download of the script.
For a direct access to the full set of data, select "Show ALL entries" in the upper left corner of the webpage.
