# Supplementary figures and images for: Identification of new signalling peptides through a genome-wide survey of 250 fungal secretomes
Source: BMC Genomics. 2019 Jan 18;20:64. doi: 10.1186/s12864-018-5414-2 (PMC6339444; doi:10.1186/s12864-018-5414-2)

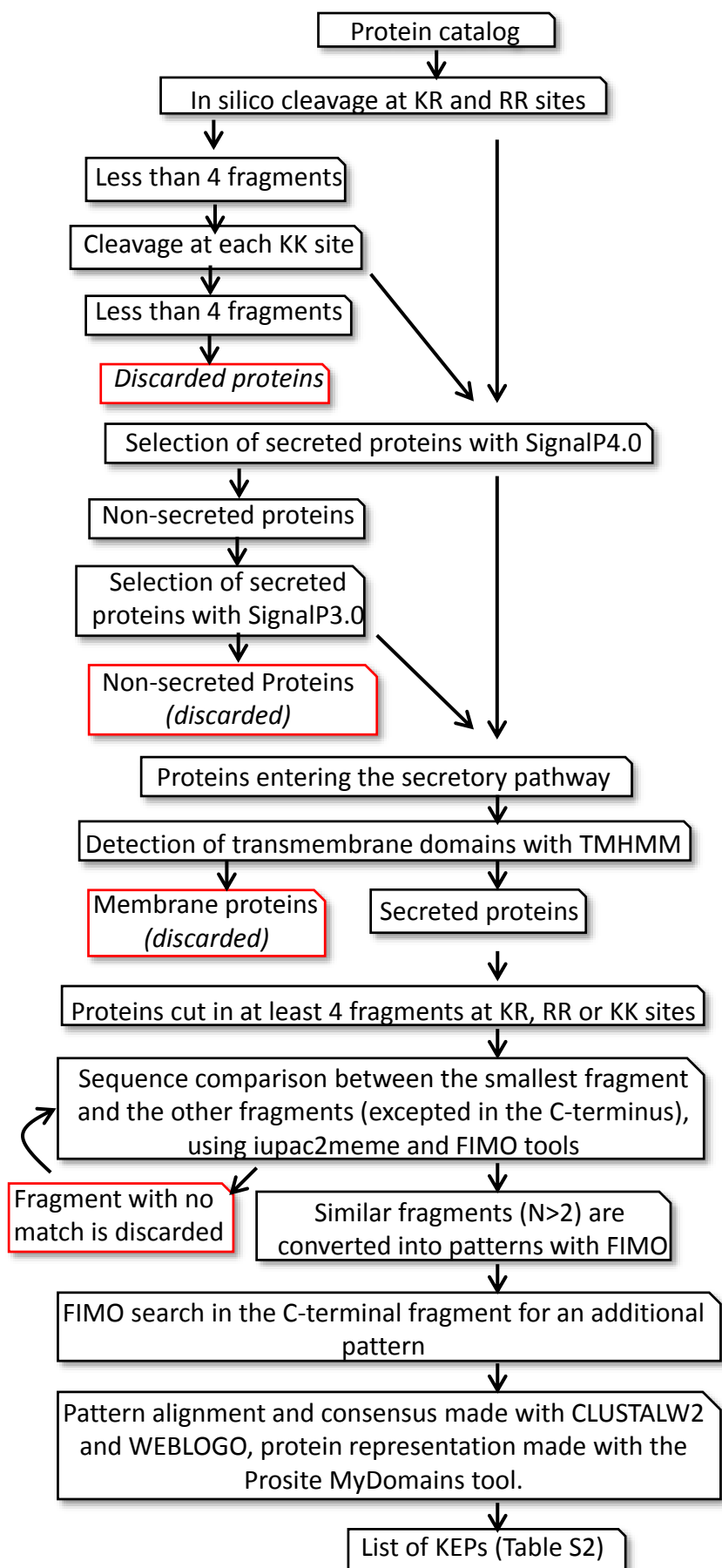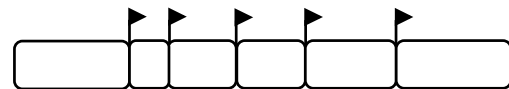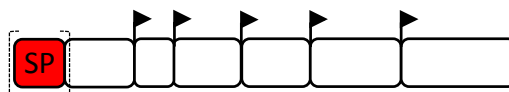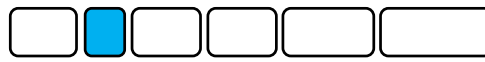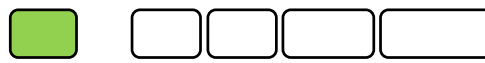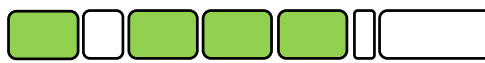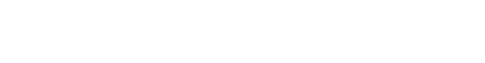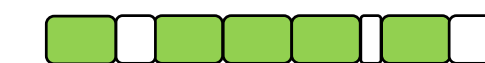

Figure S1

Supplement: Supplementary file 4 — Figure S1. Scheme depicting the informatic pipeline used to identify KEX2 processed repeat proteins (KEPs). (PDF 212 kb) [file 12864_2018_5414_MOESM4_ESM.pdf]
